# UNRES-GPU for physics-based coarse-grained simulations of protein systems at biological time- and size-scales

**DOI:** 10.1093/bioinformatics/btad391

**Published:** 2023-06-20

**Authors:** Krzysztof M Ocetkiewicz, Cezary Czaplewski, Henryk Krawczyk, Agnieszka G Lipska, Adam Liwo, Jerzy Proficz, Adam K Sieradzan, Paweł Czarnul

**Affiliations:** Centre of Informatics Tricity Academic Supercomputer and Network (CI TASK), Gdańsk University of Technology, Fahrenheit Union of Universities in Gdańsk, Gdańsk 80-233, Poland; Centre of Informatics Tricity Academic Supercomputer and Network (CI TASK), Gdańsk University of Technology, Fahrenheit Union of Universities in Gdańsk, Gdańsk 80-233, Poland; Faculty of Chemistry, University of Gdańsk, Fahrenheit Union of Universities in Gdańsk, Gdańsk 80-309, Poland; Centre of Informatics Tricity Academic Supercomputer and Network (CI TASK), Gdańsk University of Technology, Fahrenheit Union of Universities in Gdańsk, Gdańsk 80-233, Poland; Faculty of Electronics, Telecommunications and Informatics, Gdańsk University of Technology, Fahrenheit Union of Universities in Gdańsk, Gdańsk 80-233, Poland; Centre of Informatics Tricity Academic Supercomputer and Network (CI TASK), Gdańsk University of Technology, Fahrenheit Union of Universities in Gdańsk, Gdańsk 80-233, Poland; Centre of Informatics Tricity Academic Supercomputer and Network (CI TASK), Gdańsk University of Technology, Fahrenheit Union of Universities in Gdańsk, Gdańsk 80-233, Poland; Faculty of Chemistry, University of Gdańsk, Fahrenheit Union of Universities in Gdańsk, Gdańsk 80-309, Poland; Centre of Informatics Tricity Academic Supercomputer and Network (CI TASK), Gdańsk University of Technology, Fahrenheit Union of Universities in Gdańsk, Gdańsk 80-233, Poland; Centre of Informatics Tricity Academic Supercomputer and Network (CI TASK), Gdańsk University of Technology, Fahrenheit Union of Universities in Gdańsk, Gdańsk 80-233, Poland; Faculty of Chemistry, University of Gdańsk, Fahrenheit Union of Universities in Gdańsk, Gdańsk 80-309, Poland; Faculty of Electronics, Telecommunications and Informatics, Gdańsk University of Technology, Fahrenheit Union of Universities in Gdańsk, Gdańsk 80-233, Poland

## Abstract

**Summary:**

The UNited RESisdue (UNRES) package for coarse-grained simulations, which has recently been optimized to treat large protein systems, has been implemented on Graphical Processor Units (GPUs). An over 100-time speed-up of the GPU code (run on an NVIDIA A100) with respect to the sequential code and an 8.5 speed-up with respect to the parallel Open Multi-Processing (OpenMP) code (run on 32 cores of 2 AMD EPYC 7313 Central Processor Units (CPUs)) has been achieved for large proteins (with size over 10 000 residues). Due to the averaging over the fine-grain degrees of freedom, 1 time unit of UNRES simulations is equivalent to about 1000 time units of laboratory time; therefore, millisecond time scale of large protein systems can be reached with the UNRES-GPU code.

**Availability and implementation:**

The source code of UNRES-GPU along with the benchmarks used for tests is available at https://projects.task.gda.pl/eurohpcpl-public/unres.

## 1 Introduction

Coarse-grained (CG) approaches to molecular simulations enable us to treat systems at time- and size-scales by orders of magnitude larger compared with all-atom simulations (Kmiecik *et al.* 2016). Physics-based CG approaches have the advantage of being able to model structure, interactions, and dynamics. Yet treatment of large systems requires efficient use of large computational resources even at the CG level.

In our earlier work, we ported our UNited RESisdue (UNRES) program for physics-based CG simulations of protein systems to massively parallel architecture (Liwo *et al.* 2010; Lubecka *et al.* 2018), by using Message Passage Interface (MPI) and, very recently, MPI and Open Multi-Processing (OpenMP) (Sieradzan *et al.* 2023). With that last implementation, we have been able to simulate effectively microsecond-scale dynamics of about 150 000 residue protein systems in days of wall-clock time with a 24-core Central Processor Unit (CPU) per trajectory. In this work, we extended the parallelization to include Graphical Processor Units (GPUs).

Using GPUs has already been tried successfully with other CG software. For example, a 30-fold speed-up has been reached (for proteins with more than 2000 residues) with the GPU implementation of the Associative Memory, Water Mediated, Structure and Energy Model (AWSEM) model compared with the single-core CPU (NVIDIA V100 and Intel Xeon CPU E5-2650 v2) (Lu *et al.* 2021). GPU implementation of the CG MARTINI code, known as ddcMD resulted in a speed-up up to 278 with respect to a single CPU core using NVIDIA V100 and Intel Xeon E5-2695 v4, respectively (Zhang *et al.* 2020). GROMACS (Abraham *et al.* 2015) is the standard code for MARTINI simulations, but in contrast to ddcMD it employs GPU only for the most performance-critical calculations. Both GROMACS and ddcMD have a similar throughput for calculations utilizing single GPU, but ddcMD only requires a single CPU core per simulation.

Compared with other physics-based models, UNRES has two advantages which stem from rigorous derivation of the effective energy function from the potential of mean force (PMF) of CG protein systems (Liwo *et al.* 2001; Sieradzan *et al.* 2017). The first one is high degree of coarse-graining, this reducing computational cost. The second one is its ability to model regular structures without introducing heuristic-based terms or restraints. It should be noted that UNRES is tested on a regular basis in the Community Wide Experiments on the Critical Assessment of Techniques for Protein Structure Prediction (CASP) and Critical Assessment of PRediction of Interactions (CAPRI) experiments, being able to predict the structures of large proteins and protein complexes at high resolution in bioinformatics-assisted mode and of medium-size proteins at medium resolution in the *ab initio* mode (Antoniak *et al.* 2021).

## 2 Materials and methods

In the UNRES model, each polypeptide chain is represented as a sequence of C^*α*^ atoms linked by virtual bonds, with united peptide groups (**p**), each located in the middle between two consecutive C^*α*^s, and united side chains (**SC**), each attached to the respective C^*α*^ point with a virtual bond (Liwo *et al.* 2014) (Supplementary Fig. S1). The C^*α*^s only assist in geometry definition, while the **p**s and the **SC**s are interaction sites. The UNRES force field is defined on a physical basis, as the PMF of a system approximated by a truncated series of Kubo cluster cumulants (Kubo 1962; Liwo *et al.* 2001), which are identified with the respective energy terms (Supplementary Equation S1). Owing to our recently developed scale-consistent theory (Sieradzan *et al.* 2017), the effective energy function captures the correct orientation dependence of site–site interaction energies and their local-structure context. Moreover, the UNRES effective energy depends on temperature (Liwo *et al.* 2007), thus reflecting the fact that it is a PMF.

Conformational search with UNRES is carried out with Langevin or Berendsen molecular dynamics (MD) (Khalili *et al.* 2005) and its (multiplexed) replica-exchange ((M)REMD) (Hansmann 1997; Rhee and Pande 2003; Czaplewski *et al.* 2009) variants. In this work, the speed-up tests were carried out for canonical (NVT, Berendsen thermostat) MD simulations.

The MPI and OpenMP parallelization of UNRES has been described in detail in our recent paper (Sieradzan *et al.* 2023). Therefore, below we only outline the parallelization scheme and describe porting to GPU introduced in this work. The functions of UNRES currently enabled on GPU are summarized in Section S4 of Supplementary Material.

### 2.1 Two-grain parallelization scheme

The resources available to a compute job are divided between the *coarse-grained* (CG) and *fine-grained* (FG) tasks. A CG task handles a single structure (typically an MD trajectory). For multi-trajectory canonical simulations, the CG tasks synchronize only at the end of the run, while for REMD and MREMD simulations they communicate every replica-exchange interval. One CG task is assigned a master to govern the whole parallel job. Each CG task governs FG processes (MPI), threads (OpenMP or GPU).

### 2.2 CPU-based energy and force parallelization

In UNRES all interactions, including multibody interactions are effectively expressed as pairwise terms. A distance cut-off is applied and Verlet-like lists of interactions (Sieradzan *et al.* 2023) are constructed and distributed between FG tasks/threads subject to the load-balance condition. The distribution of interactions is executed bearing in mind that the space is not uniformly filled with interaction sites. Each FG task/thread carries its share of computations and the summary results are gathered at the respective CG task. For MPI, collective communication is used to distribute the work and gather results.

### 2.3 Extension of energy and force parallelization with GPU

The main loop of computations is designed as follows:

1: prepare MD step;

2: **if** interaction lists needs rebuild **then**

3:  tune the Verlet buffer size;

4:  rebuild lists of interactions;

5: **end if**

6: calculate all energy components and gradients;

7: if necessary, reduce the time step and **go to** 6;

8: complete MD step;

The Verlet buffer size is tuned to minimize the execution time by changing it by a small amount every few list rebuilds. Currently, it is changed every eight rebuilds by ±0.1 Å (while the interaction cut-off range is usually 25 Å). The direction of change is reversed if the average iteration time has increased or maintained otherwise. In the GPU path of computations, all these steps, except for the buffer size tuning, are executed on a GPU. This way, there is almost no need for exchanging data between the CPU and the GPU and only the following few scalar variables have to be passed from the GPU to the CPU in each iteration: a decision variable (indicating if list rebuilding is needed), the number of interactions (if lists have actually been rebuilt), and maximum acceleration (to determine if the time step must be reduced). For numerical stability, double precision (FP64) must be used on GPU.

Our parallel GPU code benefits from optimizations such as GPU shared memory parallel reduction and, additionally, gains approx. 12% from using multiple Compute Unified Device Architecture (CUDA) streams (Czarnul 2020) for parallel execution of CUDA kernels. The code makes use of events and CUDA stream synchronization for maintaining dependencies of a workflow within each iteration.

## 3 Results

**Figure 1.**
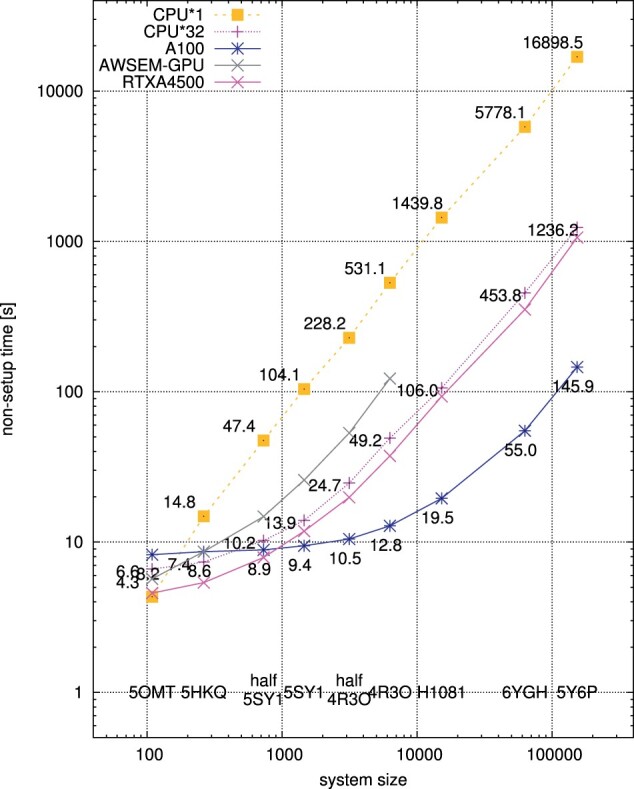
Plots of the mean non-setup time (seconds) versus system size (the number of residues). Logarithmic scale is used in both axes. Actual values are shown at each data point. Yellow symbols and line: a single CPU core, magenta symbols and line: single node (32 CPU cores), blue/pink symbols and line: a single A100/RTX A4500 GPU, grey symbols and line: AWSEM-GPU (A100).

Our parallelized CUDA code led to significant speed-ups over the CPU OpenMP version. In Fig. 1, we visualized the average (out of 3) execution times for input datasets of various sizes measured on a modern workstation with 2× AMD EPYC 7313 CPUs @3.0 GHz (2 × 16 physical cores), 8× NVIDIA A100 40GB GPU, and 4TB RAM, using Intel Parallel Studio XE 2020.4 and NVIDIA CUDA Toolkit 11.4.

Our GPU accelerated code runs on one NVIDIA A100 and one A4500 have been compared against CPU runs using 32 threads as well as using 1 thread for reference. The GPU version running on the A100 achieves the speed-ups of 8.25 and 8.47 against the 32-threaded OpenMP CPU version as well as 105.1 and 115.8 against the sequential CPU code running on a single CPU core, for the two largest systems, respectively. The GPU code on the much cheaper A4500 still outperforms the parallel CPU version. This comparison is justified by using (1) the same generation GPU and CPU and (2) the compute devices with comparable Thermal Design Power values—400 W for the A100 GPU and 310 W for 2 CPUs.

From Fig. 1, we can see that, due to the thread creation/synchronization overheads, the OpenMP version outperforms the sequential code for systems with sizes larger than approx. 200 while the GPU version outperforms the OpenMP code for systems with sizes larger than approx. 500 residues. The latter stems from host-GPU(device) communication latencies. Furthermore, the GPU code shows its full potential for systems with sizes larger than approx. 10 000 due to engaging a large number of GPU threads and increasing computations/overheads time ratios. Our version of the code also outperforms the OPEN-AWSEM GPU code (Fig. 1). Detailed numerical data can be found in Supplementary Tables S1–S4.

## Supplementary Material

btad391_Supplementary_Data
