# Supplementary material for: UNRES-GPU for physics-based coarse-grained simulations of protein systems at biological time- and size-scales
Source: Bioinformatics. 2023 Jun 20;39(6):btad391. doi: 10.1093/bioinformatics/btad391 (PMC10307937; doi:10.1093/bioinformatics/btad391)
Supplement: btad391_Supplementary_Data [file btad391_supplementary_data.zip › supplementary_file.pdf]

Supplementary Material

UNRES-GPU for Physics-Based Coarse-Grained Simulations of Protein Systems at Biological Time- and Size-Scales

Krzysztof M. Ocetkiewicz, Cezary Czaplewski, Henryk Krawczyk, Agnieszka G. Lipska, Adam Liwo, Jerzy Proficz, Adam K. Sieradzan, Paweł Czarnul

1 UNRES model

A schematic picture of the UNRES model is shown in Figure S1.

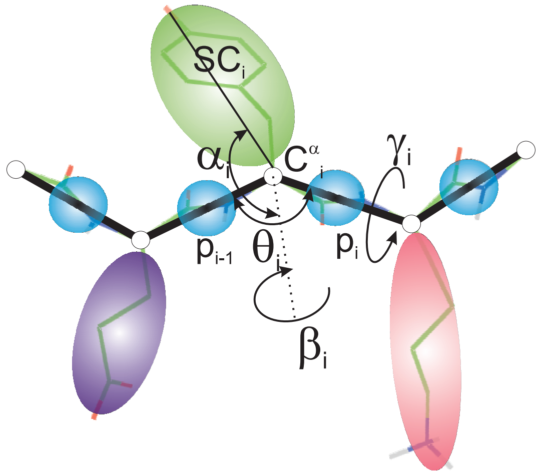

Figure S1: UNRES model of polypeptide chains. The interaction sites are united peptide groups located between the consecutive α-carbon atoms (light-blue spheres) and united side chains attached to the α-carbon atoms (spheroids with different colors and dimensions). The backbone geometry of the simplified polypeptide chain is defined by the virtual-bond angles θ and the virtual-bond-dihedral angles γ. The local geometry of the ith side-chain center is defined by the polar angle α_i and the azimuth angle β_i. Reproduced with permission from Zaborowski et al., J. Chem. Inf. Model., 55, 2050 (2015). Copyright 2015 American Chemical Society.

The UNRES energy function is expressed by equation (S1).

$$
\begin{aligned}
U &= w_{SC} \sum_{i<j} U_{SC_iSC_j} + w_{SCp} \sum_{i \neq j} U_{SC_ip_j} \\
&+ w_{pp}^{VDW} \sum_{i<j-1} U_{p_ip_j}^{VDW} + w_{pp}^{el} f_2(T) \sum_{i<j-1} U_{p_ip_j}^{el} \\
&+ w_{tor} f_2(T) \sum_i U_{tor}(\gamma_i, \theta_i, \theta_{i+1}) + w_b \sum_i U_b(\theta_i) + w_{rot} \sum_i U_{rot}(\theta_i, \hat{\mathbf{r}}_{SC_i}) \\
&+ w_{bond} \sum_i U_{bond}(d_i) + w_{ssbond} \sum_i U_{ssbond}(d_i^{SS}) \\
&+ w_{corr}^{(3)} f_3(T) \sum_{i<j-1} U_{corr;ij}^{(3)} + w_{turn}^{(3)} f_3(T) \sum_i U_{turn;i}^{(3)}
\end{aligned}
\tag{S1}
$$

where the terms $U_{SC_iSC_j}$ are sidechain-sidechain interaction energies, $U_{SC_ip_j}$ are excluded-volume potentials accounting for the interactions between united side chains and united peptide groups, $U_{p_ip_j}^{VDW}$ (with spherical symmetry) and $U_{p_ip_j}^{el}$ (with axial symmetry) are the non-bonded and mean-field-electrostatic interaction potentials of united peptide groups, U_{bond}, are the bond-deformation potentials and d_i is virtual-bond length, U_b

and U_{tor} are the backbone-virtual-bond-angle and the backbone-virtual-bond-torsional potentials, respectively, θ_i and γ_i denoting the virtual-bond- and virtual-bond-dihedral angles, respectively (Figure S1), U_{rot} are the side-chain-rotamer potentials, in which $\hat{\mathbf{r}}_{SC_i}$ denotes the local coordinates of the unit vector pointing from C_i^α to SC_i, while $U_{corr}^{(3)}$ and $U_{turn}^{(3)}$ are multibody terms that account for the coupling of the backbone-local and backbone-electrostatic interactions. U_{ssbond} denotes the terms that account for the energetics of disulfide bonds.

The factors $f_n(T)$ account for the dependence of the force-field terms that correspond to higher-order terms in the Kubo cluster-cumulant expansion on temperature [1], as given by Equation (S2).

$$f_n(T) = \frac{\ln\left[\exp(1) + \exp(-1)\right]}{\ln\left\{\exp\left[(T/T_\circ)^{n-1}\right] + \exp\left[-(T/T_\circ)^{n-1}\right]\right\}} \tag{S2}$$

where $T_\circ = 300$ K.

The ws are the weights of the energy terms and have been determined, along with some other parameters, by maximum-likelihood calibration of the force field [2]. The variant of UNRES used in this work has been termed NEWCT-9P [2].

2 Detailed results

Tables S1, S2, S3 and S4 present non-setup times and their most expensive components (in seconds) for 10,000-step single-trajectory canonical MD runs with UNRES using the A100 GPU, the A4500 GPU, 32 CPU threads and 1 CPU thread respectively. The timings of the energy/force components obtained with GPU are estimates of the active computing times of these components. The reason for this is that these values are total times during which a component was being scheduled on a GPU, i.e. it is the time difference between two events: one scheduled before all component's kernels and one scheduled after all these kernels were completed. Consequently, since all components were calculated in parallel and shared computational resources, the sum of these times exceed the total run time. Accurate timing of active computation of energy components with GPU would result in a significant performance deterioration.

Table S5 presents non-setup times for temperature annealing from 800K to 200K for 10,000-step single-trajectory runs with AWSEM using GPU.

Table S1: Results for the A100 GPU

System size	*mean [s]*	*min [s]*	*max [s]*
5OMT 109	8.24	8.21	8.28
5HKQ 263	8.63	8.60	8.64
5SY1 729	8.87	8.86	8.88
5SY1 1458	9.45	9.40	9.50
4R30 3143	10.48	10.44	10.54
4R3O 6286	12.81	12.73	12.95
H1081 15200	19.54	19.51	19.56
6YGH 62880	55.01	54.81	55.27
5Y6P 153243	145.91	145.84	145.96

$U_{SC_iSC_j}$			$U_{SC_ip_j}$			$U_{p_ip_j}$ and $U_{corr}^{(3)}$			lists		
mean [s]	*min [s]*	*max [s]*	*mean [s]*	*min [s]*	*max [s]*	*mean [s]*	*min [s]*	*max [s]*	*mean [s]*	*min [s]*	*max [s]*
0.28	0.28	0.30	0.62	0.62	0.62	1.16	1.16	1.17	1.13	1.10	1.15
0.35	0.34	0.37	0.64	0.64	0.65	1.22	1.22	1.23	1.33	1.30	1.34
0.38	0.37	0.38	0.67	0.67	0.67	1.23	1.23	1.24	1.38	1.37	1.40
0.54	0.53	0.58	0.82	0.79	0.87	1.42	1.41	1.45	1.56	1.53	1.58
0.81	0.80	0.81	1.14	1.13	1.14	1.86	1.85	1.88	2.13	2.07	2.18
1.82	1.77	1.85	2.13	2.10	2.18	3.58	3.56	3.63	2.57	2.42	2.75
4.88	4.83	4.93	5.58	5.47	5.76	8.68	8.62	8.76	3.69	3.57	3.80
17.61	17.47	17.74	23.24	23.20	23.34	34.39	34.34	34.44	9.99	9.77	10.16
43.70	43.45	44.11	68.71	68.48	69.09	103.03	102.56	103.83	25.73	24.69	26.32

Table S2: Results for the A4500 GPU

System size	*mean [s]*	*min [s]*	*max [s]*
5OMT 109	4.61	4.59	4.63
5HKQ 263	5.39	5.36	5.41
5SY1 729	7.89	7.88	7.90
5SY1 1458	11.75	11.70	11.79
4R30 3143	19.72	19.47	19.89
4R3O 6286	37.70	37.62	37.83
H1081 15200	92.74	92.67	92.80
6YGH 62880	351.16	351.00	351.25
5Y6P 153243	1061.42	1060.95	1062.12

$U_{SC_iSC_j}$			$U_{SC_ip_j}$			$U_{p_ip_j}$ and $U^{(3)}_{corr}$			lists		
mean [s]	*min [s]*	*max [s]*	*mean [s]*	*min [s]*	*max [s]*	*mean [s]*	*min [s]*	*max [s]*	*mean [s]*	*min [s]*	*max [s]*
0.31	0.31	0.31	0.40	0.40	0.40	0.67	0.67	0.68	0.60	0.58	0.62
0.80	0.77	0.83	0.87	0.84	0.89	1.27	1.25	1.28	0.74	0.72	0.77
2.52	2.51	2.54	2.46	2.42	2.50	3.27	3.26	3.27	0.88	0.85	0.90
5.69	5.62	5.79	5.61	5.48	5.71	6.72	6.66	6.78	1.20	1.18	1.22
11.43	11.37	11.49	8.68	8.62	8.71	13.92	13.77	14.01	2.19	1.86	2.52
20.76	20.59	20.86	19.55	19.49	19.61	30.23	30.03	30.34	3.86	3.52	4.31
42.94	42.87	43.06	77.28	77.11	77.43	80.28	80.14	80.45	8.77	8.59	8.87
146.30	146.17	146.46	262.73	261.00	265.19	308.52	307.82	309.01	36.37	35.64	37.37
450.79	449.74	451.68	902.25	898.08	909.62	937.52	933.38	943.20	115.77	109.87	120.25

Table S3: Results for 32 CPU threads

System size	*mean [s]*	*min [s]*	*max [s]*
5OMT 109	6.61	6.45	6.72
5HKQ 263	7.36	7.27	7.47
5SY1 729	10.23	9.97	10.39
5SY1 1458	13.92	13.70	14.13
4R3O 3143	24.68	24.35	25.29
4R3O 6286	49.20	46.68	50.64
H1081 15200	105.95	105.36	106.34
6YGH 62880	453.83	453.33	454.70
5Y6P 153243	1236.16	1225.23	1254.17

$U_{SC_iSC_j}$			$U_{SC_ip_j}$			$U_{p_ip_j}$ and $U^{(3)}_{corr}$			lists		
mean [s]	*min [s]*	*max [s]*	*mean [s]*	*min [s]*	*max [s]*	*mean [s]*	*min [s]*	*max [s]*	*mean [s]*	*min [s]*	*max [s]*
0.40	0.37	0.44	0.14	0.14	0.15	0.83	0.77	0.91	2.37	2.17	2.55
0.67	0.65	0.69	0.20	0.20	0.20	1.14	1.10	1.17	2.24	2.09	2.33
1.39	1.33	1.43	0.38	0.37	0.40	1.99	1.89	2.04	2.16	1.66	2.65
2.48	2.44	2.51	0.71	0.69	0.72	3.20	3.13	3.25	2.31	2.17	2.40
5.26	5.17	5.37	1.40	1.38	1.41	6.44	6.34	6.49	2.70	2.41	3.01
11.35	10.96	11.81	3.27	3.22	3.30	13.45	13.00	13.87	5.83	4.99	6.64
27.42	27.31	27.62	8.41	8.31	8.56	30.41	30.36	30.49	10.46	9.90	10.84
116.40	115.82	117.03	36.26	35.49	36.71	140.84	139.86	141.72	40.75	39.55	43.08
343.06	335.58	349.55	96.59	94.39	100.00	398.94	394.72	405.13	115.72	114.71	116.38

Table S4: Results for 1 CPU thread

System size	*mean [s]*	*min [s]*	*max [s]*
5OMT 109	4.33	4.17	4.45
5HKQ 263	14.84	14.81	14.86
5SY1 729	47.37	47.34	47.39
5SY1 1458	104.12	103.74	104.40
4R3O 3143	228.23	228.09	228.52
4R3O 6286	531.07	529.82	531.72
H1081 15200	1439.85	1438.85	1440.67
6YGH 62880	5778.11	5771.18	5783.32
5Y6P 153243	16898.47	16888.40	16918.20

$U_{SC_iSC_j}$			$U_{SC_ip_j}$			$U_{p_ip_j}$ and $U_{corr}^{(3)}$			lists		
mean *[s]*	*min* *[s]*	*max* *[s]*	*mean* *[s]*	*min* *[s]*	*max* *[s]*	*mean* *[s]*	*min* *[s]*	*max* *[s]*	*mean* *[s]*	*min* *[s]*	*max* *[s]*
1.46	1.40	1.48	0.48	0.46	0.51	1.42	1.36	1.46	0.25	0.24	0.26
5.58	5.55	5.59	2.21	2.19	2.24	5.11	5.10	5.12	0.60	0.57	0.63
18.21	18.18	18.23	7.14	7.13	7.15	16.09	16.07	16.11	2.67	2.66	2.68
39.60	39.21	39.82	15.63	15.48	15.71	34.94	34.69	35.16	7.65	7.42	8.10
85.55	85.40	85.69	36.14	35.84	36.39	77.50	77.25	77.95	15.68	15.24	16.21
198.98	197.14	200.93	84.56	83.76	85.90	177.51	176.07	179.05	42.09	38.07	46.75
548.44	545.39	551.94	223.03	222.21	223.70	473.64	472.87	474.27	121.26	116.97	126.12
2181.1	2170.5	2193.0	861.6	859.1	865.9	1859.6	1854.9	1864.7	540.6	525.2	559.2
6496.5	6460.4	6528.9	2487.2	2478.6	2500.6	5366.4	5342.6	5382.1	1696.0	1654.3	1750.2

Table S5: Times of the simulations for AWSEM force field with use of OPENAWSEM run on the A100 GPU

System size	*mean [s]*	*min [s]*	*max [s]*
5OMT 109	5.69	5.47	6.11
5HKQ 263	8.63	8.45	8.97
5SY1 729	14.79	14.76	14.81
5SY1 1458	25.84	25.63	26.17
4R3O 3143	53.11	52.96	53.32
4R3O 6286	122.26	121.87	122.89

3 Hardware and software setup

Hardware used:

server with 2 × AMD EPYC 7313 CPUs @3.0GHz (2×16 physical cores), 8 × NVIDIA A100 40GB GPU and 4TB RAM.

server with 2 × Intel Xeon Silver 4316 CPU @2.3GHz (2×20 physical cores), NVIDIA RTX A4500 20GB GPU and 256MB RAM

Software used:

Intel Parallel Studio XE 2020.4 as well as Cuda compilation tools, release 11.4, V11.4.120.

Compilation commands:

```
IFORT: -qopenmp -O3 -ipo -march=core-avx2 -fimf-precision=high -prec-div -prec-sqrt
NVCC: -O3 -arch=sm_80 --std c++17 -rdc=true
```

The code used in tests was targeted for CUDA Compute Capability 8.0.

4 UNRES features CPU/GPU

Table S6: Features (un)available on CPU/GPU: Constant number of particles, constant volume and constant energy(NVE); Berendsen thermostat; Langevin thermostat; Minimization; Restraints; Steered-molecular dynamics (SMD); Variable time step (VTS); Adaptive multiple times step (AMTS); Multiplexed replica exchange molecular dynamics (MREMD); Dynamic formation/breaking disulfide bonds (Dyn_{ss}).

Feature	*CPU*	*GPU*
NVE	✓	✓
Berendsen thermostat	✓	✓
Langevin thermostat	✓	✓
Minimization	✓	*
Restraints	✓	✓
SMD	✓	✓
VTS	✓	✓
AMTS	✓	×
MREMD	✓	×
Dyn_{ss}	✓	✓

* Minimization before MD is preformed by CPU.

5 Implementation considerations

5.1 Data model

Interactions to be processed are kept in two lists per interaction kind. One list holds pairs of interacting sites (e.g. (i, j), the other one keeps interactions as a range of sites interacting with a given site (e.g. (i, j_{first}, j_{last})). There is also a minimal range length: a sequence of consecutive interactions is not considered a range if it is shorter than such a minimum and these interactions are inserted into the pairs list instead. The same format is used in a CPU path for storing interactions. However, the goals of these two lists on the GPU and the CPU are opposite. On the CPU we want as many interactions as possible in the ranges form. Processing the ranges of interactions takes less time than processing individual pairs (considering the time used per single interaction) due to the relatively low performance of the CPU scatter/gather instructions. Even the ranges as short as four sites are worth storing as a range. On the other hand, on the GPU side, the pairs form is preferred. Ranges are more difficult to process for the GPU due to the highly parallel nature of its computational resources and strict rules governing scheduling work to individual computing resources. Therefore, the minimum range length is set to 64. The list of ranges on a GPU is more of a memory usage limiting tool. As long as memory allows, the pairs list is preferred. However, if one wished to use a very large cutoff length, the list of pairs would easily exhaust the available memory. During execution, every pair is assigned to a separate GPU thread, while every range is handled by a single GPU warp, i.e. a group of 32 threads. Energy terms that do not use lists of interactions are served by a single GPU thread per residue per energy term.

In an example MD run of 5Y6P system (154975 residues) the recorded number of symmetric interactions (P-P and SC-SC) per residue for every such interaction were about 125 pairs at the start of simulation, with an initial buffer width of 0.5Å, to about 165 at the end of simulation, when the buffer width stabilised near 2.0Å. The average number of ranges per residue was $0.03 \ldots 0.16$ (depending on the buffer width) with an average range length of 82. For non-symmetric interactions, i.e. SC-P, these numbers are essentially doubled: about 255 pairs and 0.15 ranges per residue with 0.5Å cutoff up to 340 pairs and 0.3 ranges per residue, again with an average length of about 81. On top of that, for every site up to 14 additional energy contributions are calculated, that reflect the terms of the UNRES energy function or the bounds imposed on the system.

5.2 Work scheduling

The GPU workload is split into multiple kernels that, in turn, are scheduled into multiple streams. This is done to maximize GPU utilization. Energy terms are mostly independent and, therefore, can be calculated in parallel with little synchronization. Every energy component has one or more dedicated kernels and one or more dedicated streams, depending on the type of calculations. For example, seven kernels are used to handle electrostatic interactions: one for individual pairs, one for ranges of interactions, two for consecutive sites in polypeptide chain and three kernels for data preparation and postprocessing. These kernels are scheduled into four streams: after preprocessing finishes, the four computing kernels (pairs, ranges and two consecutive) run in parallel in separate streams. Finally, the postprocessing kernels wait for the computing kernels to finish and execute postprocessing instructions in a sequence. On the other hand, virtual-bond-angle energy needs only one kernel and one stream. However, since it is a different stream, if GPU resources allow, electrostatic energy/forces and virtual-bond-angle energy/forces can be computed in parallel. The total number of kernels executed in each MD step depends on the exact energy contributions enabled, but is about 50 in a typical step and about 60 in a step that requires rebuilding lists of interactions.

5.3 Precision

The calculations on a GPU are performed using FP64 numbers. This means that a GPU with at least CUDA Compute Capability 6.x is required since it is the first version to support atomic updates of FP64 values. FP64 performance on many commodity GPUs is significantly reduced compared to datacenter targeted GPUs. For example, NVIDIA A100 offers FP64 performance that is 1/2 of its FP32 performance while most consumer GPUs offer 1/32 or even 1/64 FP64 to FP32 ratio. While the code has been verified to run even on an NVIDIA GT 1030, it is not the optimal hardware to run UNRES.

6 Possible applications

The implementation of UNRES on the GPU's allows us to run simulations much faster than previously. For instance, the MD simulations of virus-like particles of human noroviruses with the use of an optimized CPU-based approach took 21–25 wall-clock days (with two Intel® Xeon® E5 v3 @ 2,3 GHz, 12-core (Haswell) processors) to get a 30 ns trajectory [3]. The use of the GPU version (using NVIDIA A100) allows us to get the same time scale within 24 hours. The system was composed of 180 chains giving approximately $97,000$

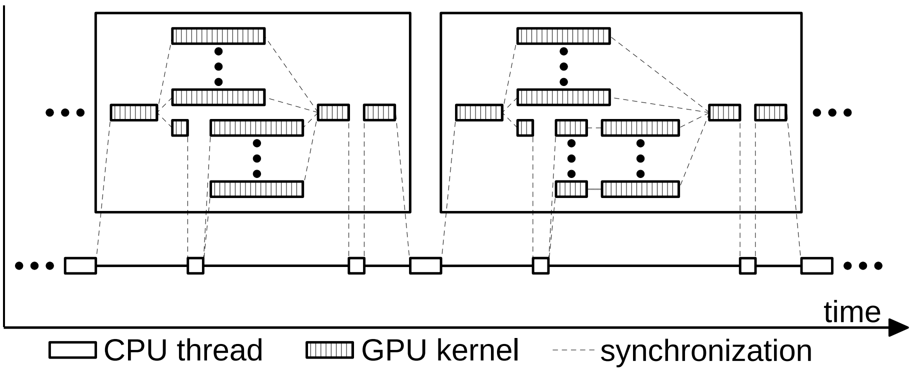

Figure S2: Schematic work partitioning between the CPU and the GPU during MD calculations. During an MD step, synchronization (dashed lines) between the CPU and the GPU is needed only between consecutive steps, after checking if lists rebuild is needed and near the end of step, when the decision whether to reduce time step is made. When interaction lists must be rebuilt (the second part of the diagram) additional kernels must be scheduled to build new lists, compared to a typical step (the first part of the diagram).

amino acid sidechains. This speed-up allows us to examine more strains or to use this tool to predict the dynamic properties of the human-induced mutations of virus-like particles on the way of designing the vaccine components.

Another example are the simulations of microtubulin (MT). MTs are polymers of $\alpha\beta$-tubulin heterodimers that are part of the cytoskeleton and play important roles in the eucaryotic cell eg.: maintaining cell shape, and motility. The starting model was built based on the deacylated microtubulin structure from PDB database (PDB ID: 6O2S [4]). The system contains 520 chains, and 233,220 amino-acid residues total (Figure S3 A). The wall-clock time was 42 h with the GPU-based UNRES version on NVIDIA A100. It would take approximately 40 days with the CPU (24 cores).

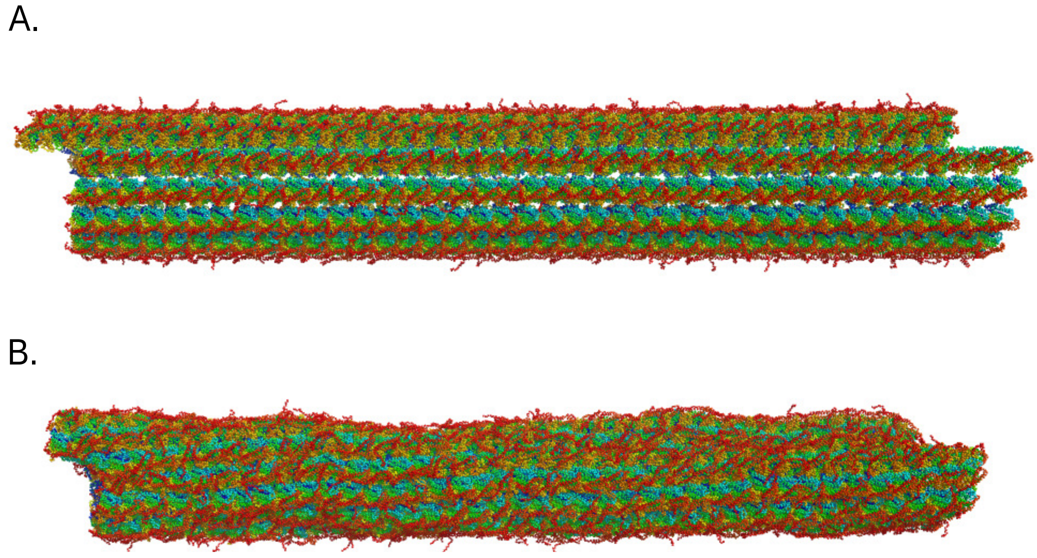

Figure S3: The initial (A) and final (B) microtubulin models. Only C-α atoms are shown

References

[1] A. Liwo, M. Khalili, C. Czaplewski, S. Kalinowski, S. Ołdziej, K. Wachucik, and H. A. Scheraga. Modification and optimization of the united-residue (UNRES) potential energy function for canonical simulations. I. Temperature dependence of the effective energy function and tests of the optimization method with single training proteins. *J. Phys. Chem. B*, 111:260–285, 2007.

[2] A. Liwo, A. K. Sieradzan, A. G. Lipska, C. Czaplewski, I. Joung, W. Żmudzińska, A. Hałabis, and S. Ołdziej. A general method for the derivation of the functional forms of the effective energy terms in coarse-grained energy functions of polymers. III. Determination of scale-consistent backbone-local and correlation potentials in the UNRES force field and force-field calibration and validation. *J. Chem. Phys.*, 150:155104, 2019.

[3] A. G. Lipska, A. K. Sieradzan, C. Czaplewski, A. D. Lipińska, K. M. Ocetkiewicz, J. Proficz, P. Czarnul, H. Krawczyk, and A. Liwo. Long-time scale simulations of virus-like particles from three human-norovirus strains. *J. Comput. Chem.*, 44(16):1470–1483, 2023.

[4] L. Eshun-Wilson, R. Zhang, D. Portran, M. V. Nachury, D. B. Toso, T. Löhr, M. Vendruscolo, M. Bonomi, J. S. Fraser, and E. Nogales. Effects of α-tubulin acetylation on microtubule structure and stability. *Proc. Natl. Acad. Sci. U.S.A.*, 116(21):10366–10371, 2019.
