# Supplementary material for: UNRES-GPU for physics-based coarse-grained simulations of protein systems at biological time- and size-scales
Source: Bioinformatics. 2023 Jun 20;39(6):btad391. doi: 10.1093/bioinformatics/btad391 (PMC10307937; doi:10.1093/bioinformatics/btad391)

A.

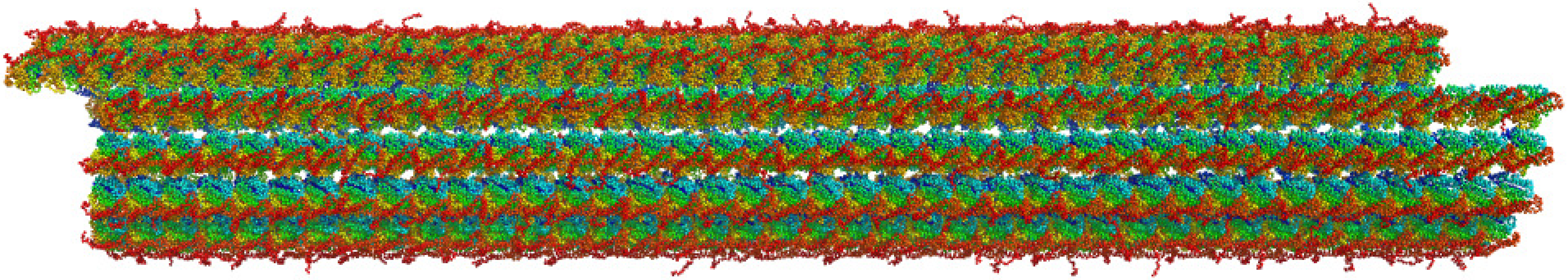

B.

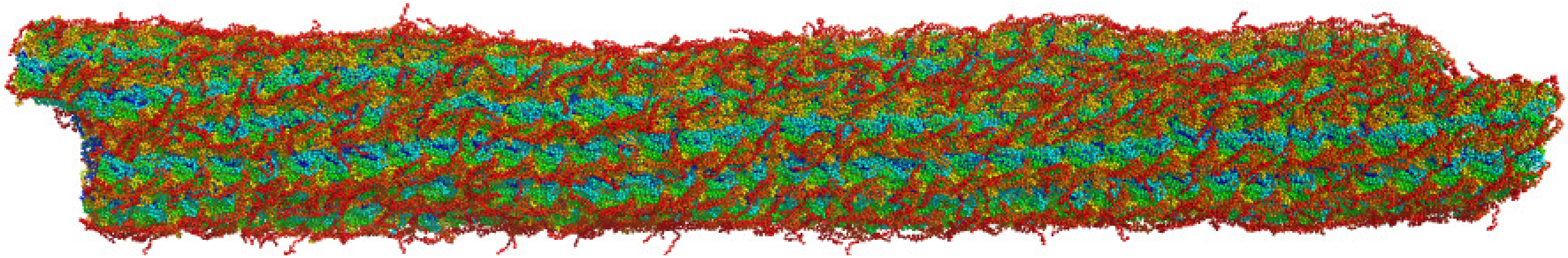

Supplement: btad391_Supplementary_Data [file btad391_supplementary_data.zip › supplementary-material-src-final-clean.zip!/supplementary-material-src/str-5MT-2-eps-converted-to.pdf]
